# Using qualitative research perspectives to inform patient engagement in research

**DOI:** 10.1186/s40900-018-0107-1

**Published:** 2018-07-06

**Authors:** Michelle Phoenix, Tram Nguyen, Stephen J. Gentles, Sandra VanderKaay, Andrea Cross, Linda Nguyen

**Affiliations:** 1 Bloorview Research Institute, Holland Bloorview Kids Rehabilitation Hospital, 150 Kilgour Road, Toronto, ON M4G 1R8 Canada; 2 CanChild, Institute for Applied Health Sciences, McMaster University, 1400 Main Street West, Room 408, Hamilton, ON L8S 1C7 Canada; 3 School of Rehabilitation Science, Institute for Applied Health Science, McMaster University, 1400 Main Street West, Room 403, Hamilton, ON L8S 1C7 Canada; 4 School of Epidemiology, Public Health, and Preventative Medicine, Alta Vista Campus, 600 peter Morand Crescent, Ottawa, ON K1G 5Z3 Canada; 5 Offord Centre For Child Studies, Department of Psychiatry and Behavioural Neurosciences, McMaster University, 1280 Main St. W., Hamilton, ON M1P 201A Canada

**Keywords:** Patient engagement in research, Qualitative research, Healthcare providers, Health research, Methodology, Rehabilitation

## Abstract

**Plain English summary:**

In Canada, and internationally, there is an increased demand for patient engagement in health care research. Patients are being involved throughout the research process in a variety of roles that extend beyond the traditional passive participant role. These practices, referred to collectively as ‘patient engagement’, have raised questions about how to engage patients in the research process. Specifically, researchers have noted a lack of theory underpinning patient engagement and are looking for guidance on how to select patients and engage patients throughout the research process. In this commentary, we draw on qualitative research perspectives to generate theoretical and methodological ideas that novice or experienced researchers can apply to facilitate patient engagement in research.

**Abstract:**

Despite the recent advancements in patient engagement in health care research, there is limited research evidence regarding the best strategies for developing and supporting research partnerships with patients and caregivers. Three particular outstanding concerns that have been reported in the literature and that we will explore in this commentary are: (i) the lack of theoretical underpinning to inform the practice of patient engagement in research; (ii) the lack of knowledge regarding how to select patients to engage in research; and (iii) the lack of clear guidance about the best methods for engaging patients in research. We draw on qualitative research perspectives to reflect on these three areas of concern and propose insights into the theory and methods that we believe are useful for engaging patients in research.

## Background

As pediatric rehabilitation researchers who have conducted qualitative research, we recognize the value of engaging patients in the research process. We use “patient” to include patients and their caregivers. The widespread use of the language of patient engagement (including the terms public-, stakeholder- and citizen-engagement) indicates that patients are no longer viewed solely as research *participants*, but rather influential *partners* throughout the research process [1].

Several benefits of engaging patients in the research process, as identified by patients and researchers, include increased relevance and applicability of research outcomes [2–4], increased patient enrollment and retention [5, 6], and increased partnership development and patient empowerment [7, 8]. There is limited research evidence, however, to indicate which strategies are best for developing research partnerships with patients, or the impact of these partnerships in improving research outcomes [5, 7, 9].

Funding agencies and researchers have shown increased interest in promoting patient engagement in research and have provided a variety of recommendations about how to engage patients in research [10, 11]. However, the evidence underlying these recommendations and the reporting of patient engagement is often insufficient to inform best practices [5, 7, 9, 12]. Following their systematic review of the literature on patient engagement in research Domecq and colleagues [5] identified three main areas of concern where guidance is needed: (i) the lack of theoretical underpinning to inform the practice of patient engagement in research; (ii) the lack of knowledge regarding how to select patients to engage in research; and (iii) the lack of clear guidance about the best methods for engaging patients in research. We framed our discussion according to the concerns presented by Domecq and colleagues [5] because their recent systematic review has been widely cited and it sparked our discussion regarding how qualitative research perspectives can provide theoretical and methodological insights to facilitate patient engagement in research.

### Main text

#### Using qualitative perspectives to inform the theoretical underpinnings of patient engagement in research

Qualitative research seeks to understand people’s experiences, and circumstances, as well as the underlying meanings shaped from people’s perspectives; thereby accepting the belief that an individuals’ experience of the world is subjective and may be different from one person to another [13]. These philosophical beliefs stand in contrast to beliefs that there is a single reality that can be tested in ways that minimize researcher bias [14]. Notably there may be a continuum and melding of these philosophical beliefs and assumptions for researchers who seek to engage patients in research.

We suggest that the theoretical paradigms that are often associated with qualitative research may offer guidance to researchers conducting research with patients. Qualitative and quantitative researchers who engage in patient-centred research should try to understand the context of patients’ lives, listen to and value patients’ experiences, and co-create meaning and ideas throughout the research process. Certain qualitative theoretical paradigms, such a social constructivism or feminist theory, may help by providing insight into how to respectfully access patient values and experiences and construct a shared meaning that reflects researcher and patient perspectives [14].

Often qualitative research employs an emergent design whereby participants’ views are used to develop and revise interview guides and categories for analysis [14]. These practices, of using patient ideas and suggestions to revise the study questions, materials, methods, or analysis can be also applied in patient engagement in research where patients’ insights can influence all aspects of the research process from study conceptualization to application of the results [15]. Employing principles of flexibility and responsiveness throughout a study allows for the integration of patient suggestions and may help to avoid tokenism, which was noted as a common pitfall when researchers retain power while involving patients in research [15, 16]. Researchers may benefit from additional training to learn how to identify social dynamics on research teams and appreciate the realities of patient experiences, while patients may benefit from training to understand content and methods to promote confidence and active engagement in research [7, 16].

Eliciting patient suggestions and perspectives requires researchers to build relationships with patients to minimize power imbalances and provide clarity regarding roles and contributions [15, 16]. This closeness with participants is commonly associated with qualitative research as researchers often invest significant time in building rapport with participants to better understand the depth and context of participant perspectives. Taking the time to interact and establish trust with patients from the start of a study can help to negotiate roles, balance power, and lead to meaningful patient collaboration [7, 17, 18]. Researchers who engage in participatory action research have established methods, histories and successes in joining citizens as equals to design and carry out research to meet the needs as identified by the citizen group in ways that enact change [19, 20].

#### Employing qualitative research approaches to select patients and address biases

There are two main ways in which patients are recruited for engaging in the research process, (i) open strategies which allow for patients to self identify and volunteer for open postings or (ii) direct invitations in which specific partners are identified and asked to participate [7]. While patients who self identify and volunteer to join a research project may be highly motivated, those selected this way may have their own personal agendas that are not reflective of the target population as a whole [5]. Although random sampling may seem a logical solution, it is not feasible in the many situations where only small number of patients are available or needed, and there is no evidence that random sampling is superior to purposeful or convenience sampling for patient engagement in research [5].

We suggest that more explicit acknowledgement of a wider range of selection approaches is needed to: (a) reflect the variety and merits of contemporary practices that researchers are using, and (b) appropriately meet the diverse objectives associated with different research stages and patient engagement activities. Accordingly, qualitative research methods are heuristically useful to broaden researchers’ menu of sampling approaches. Participant selection in qualitative research often involves purposeful sampling, prioritizing inclusion of information-rich cases from which one can learn much about issues of central importance [21]. Importantly, purposeful sampling is highly adaptable, and thus applicable to many of the varying aims of engaging patient partners. For example, in seeking input on recruitment methods, a purposeful approach is ideal to access perspectives of marginalized groups about how to maximize response rates from such difficult-to-access participants, increasing representativeness of participant samples and reducing inequity in research [22]. Furthermore, if patients were being recruited to a team to help with technical skills or creating lay summaries to support knowledge translation, purposeful approaches would also be useful for identifying and recruiting individuals best suited to meet these objectives. Specific considerations are provided by INVOLVE to help determine who should be involved in research, depending on the purpose and level of support available, and the ways to find them [23]. Moreover, the qualitative sampling literature contains additional methodological insights relevant to the goals of engaging patients in research, such as suggestions on how to purposefully sample participants to enrich rather than inhibit focus group participant interactions [24, 25]. Doria and colleagues [26] provide a fulsome comparison of focus groups used in qualitative research and discussion groups used for patient engagement noting similarities and differences in the purpose, methods and ethical considerations.

Practicing reflexivity may be particularly useful when researchers are collaborating with patients who have self-selected, or volunteered, to contribute to a research activity, as they may bring agendas that are not representative of the rest of the patient population. Although most extensively characterized in the qualitative methods literature [27], reflexivity has been practiced by qualitative, mixed methods or quantitative researchers to make their influence on the research explicit—to themselves, and often their audience. In applying reflexivity to research engagement, researchers responsible for recruiting patients to engage could themselves first reflect on the possible influences of their own and the patients’ personal biases. Once convened, researchers and patients could then be led through reflexive exercises encouraging them to reflect on personal agendas, their preconceptions on the topic at hand, whose agendas and priorities are being ignored, and how this may influence the research.

Although adopting reflexivity may lengthen the research process, it may help to achieve the key principles of patient engagement in research as identified by INVOLVE (e.g., respecting and valuing the knowledge of all team members [28]), and aligns with INVOLVE’s recommendation to make a team plan for continuous reflection throughout a project (e.g., journaling and holding periodic reflective meetings [28]). Documenting the personal commitments of the researchers and patients may increase transparency and contribute deeper thinking to inform the patient engagement process. Time and feasibility are reported challenges of patient engagement in research [5, 7], therefore it will be critical to determine the purpose and best methods for reflexivity in the context of engagement activities with consideration for project feasibility and resource allocation.

#### Using qualitative methods to inform strategies for patient engagement in research

The most commonly used methods to engage patients in the research process (i.e., focus groups, interviews, surveys and participation on advisory councils) are approaches that are often used in qualitative research [5, 20]. Qualitative researchers frequently conduct in-depth interviews with individual patients to enhance the breath, depth and richness of information on a specific topic under study [14]. In 2005, Mack and colleagues published a “field guide” outlining how to use focus groups and interviews in qualitative research to gather data and engage individuals with a wide range of expertise and experience [29]. Strategies used within these qualitative methods, such as active listening, neutral questioning, reflection and co-construction of knowledge, are applicable to patient engagement and can assist in building rapport and trust amongst patient and researcher partners. We acknowledge that patient-centred researchers who employ mixed methods or quantitative research approaches may already use these strategies to engage patients through the research process. We suggest that researchers of any tradition who wish to learn more about the strategies needed to build rapport and trust with research partners, or engage patients on research teams through focus groups or interviews, would be well served to access the vast qualitative literature that addresses these methods of engaging with patients.

### An example

To show how these ideas can be applied, we present an example of the ‘F-words’ integrated knowledge translation research program that began at *CanChild* in 2014 to disseminate and support the adoption of the ‘F-words’ in Childhood Disability [17, 30]. The ‘F-words’ (Function, Family, Fitness, Fun, Friends, and Future) are an operationalization of the World Health Organization’s International Classification of Functioning, Disability and Health (ICF) framework [31]. This project is described in published manuscripts [17, 32], however we highlight examples in each of the areas of concern discussed above as concrete illustrations of our ideas. First, the research program followed an integrated knowledge translation approach and was flexible in the parent caregivers’ roles on the team and responsive to their contributions in study design and the co-creation of knowledge translation products. Second the parent members of the research team were purposefully selected using direct invitation because they had previously shown interest in the ‘F-words’ concepts. Options were provided for level of involvement and tasks to be undertaken by parent team members. Third, qualitative research strategies such as active listening (in team meetings), reflecting back (frequent check in’s with team members and opportunities to provide feedback) and co-constructing knowledge (e.g., co-creating knowledge translation products), were used to build a successful and meaningful research partnership.

## Conclusions

We have outlined three areas where a qualitative perspective may be helpful to address previously identified concerns with patient engagement in research: its theoretical underpinnings, the selection of patients to engage, and strategies for patient engagement. We hope these points will stimulate further discussion among researchers and trainees about how qualitative paradigms and methods have the potential to facilitate the many aims of patient engagement in research. Further research will be helpful to evaluate the proposed ideas and their impact on partnerships between researchers and patients.
